# Laboratory and Point-of-Care Testing for COVID-19: A Review of Recent Developments

**DOI:** 10.7759/cureus.28530

**Published:** 2022-08-29

**Authors:** Ravi Kalia, Rishi Kaila, Payal Kahar, Deepesh Khanna

**Affiliations:** 1 Medicine, Nova Southeastern University, Dr. Kiran C. Patel College of Osteopathic Medicine, Tampa, USA; 2 Medicine, Nova Southeastern University, Dr. Kiran C. Patel College of Osteopathic Medicine, Fort Lauderdale, USA; 3 Health Sciences, Florida Gulf Coast University, Fort Myers, USA; 4 Foundational Sciences, Nova Southeastern University, Dr. Kiran C. Patel College of Osteopathic Medicine, Clearwater, USA

**Keywords:** antigen, elisa, rt-pcr, covid-19 nucleic acid testing, serial laboratory testing in covid -19, covid-19, coronavirus, coronavirus disease, point of care testing, laboratory tests

## Abstract

With the emergence of Coronavirus infection called COVID-19, testing is essential for containment and mitigation purposes. In a pandemic, control is essential to limit the spread of any virus. Initially, contact tracing was not available which ultimately led to the 2020 pandemic. However, with the development of COVID-19 rapid testing, the rate of infections has lessened and has allowed for some return to normalcy. In this review, we discuss the various antibody, antigens, and molecular tests that have been given emergency authorization (EA) from the Food and Drug Administration (FDA). Moreover, we will discuss the various point-of-care tests as well as the specificity and sensitivity that are associated with each testing kit. With appropriate testing, we can be aware of how the virus spreads and how prevalent it remains.

## Introduction and background

COVID-19, a severe respiratory syndrome, caused by a novel coronavirus, severe acute respiratory syndrome coronavirus 2 (SARS-CoV-2), led to a pandemic when it originated in Wuhan, China in 2019 and spread across the globe throughout 2020 [1,2]. SARS-CoV-2 is an RNA, positive-sense, single-stranded, enveloped beta coronavirus and shows greater than 96% identity to a known bat coronavirus [3,4]. As of July 2022, there have been 558 million cases and 6.4 million deaths worldwide associated with SARS-CoV-2 as reported by the Centers for Disease Control and Prevention (CDC) [5].

The incubation period for COVID-19 is approximately 2-14 days and frequently individuals present with fever, cough, muscle/body aches, fatigue, and shortness of breath [6]. Infected individuals may either be asymptomatic or symptomatic with mild or severe symptoms. The transmission is dependent upon direct contact, fomites, or exposure to virus droplets, especially if the exposed individual is within 6 feet away from an infected individual [6]. Some major COVID-19 complications include respiratory dysfunction, cardiovascular dysfunction, blood clotting, and ocular manifestations. Individuals can be protected against this virus by way of social distancing, wearing masks, as well as receiving the vaccine [6]. At this moment, there is four FDA emergency use authorization (EUA) approved COVID-19 vaccines on the market - Pfizer-BioNTech, Moderna, Johnson & Johnson, Oxford Astrazeneca, and Novavax - which are effective and significantly reduce the risk of severe illness [3,4,7-10].

When the pandemic was classified by WHO, researchers worldwide rushed to decode the genome and were able to formulate diagnostic protocols utilizing nucleic acid amplification techniques (NAAT). These techniques were limited to certain laboratories and were not able to give rapid results. However, soon after, commercially generated rapid assays were brought to market and individuals were either able to be tested at their local pharmacy or home within minutes. This set a new standard of testing during the pandemic. These new assays that tested for antigens or antibodies varied in their detection modality.

In a pandemic, control is essential to limit the spread of any virus. With the emergence of COVID-19 in Wuhan, contact tracing was not available which ultimately led to the 2020 pandemic. However, with the development of COVID-19 rapid testing, the rate of infections has lessened and has allowed for some return to normalcy. As these COVID-19 tests and mechanisms have not been discussed yet, in this article, we aim to guide individuals to choose the appropriate testing modality based on the overall COVID-19 situation and to highlight certain limitations as well as advantages associated with some of these testing kits [11].

## Review

Methods

Authors browsed PubMed articles using the keywords “SARS-CoV-2,” “COVID-19 Tests,” “COVID-19 Rapid tests,” “COVID-19 Antigen Tests,” “COVID-19,” “COVID-19 Antibody Tests,” “COVID-19 Diagnostic,” and “COVID-19 RT-PCR”. Additionally, the authors searched CDC, WHO, and FDA’s website for specific antigens, antibodies, nucleic acid, and point-of-care tests specific to SARS-CoV-2. On Google Scholar, we searched using the same parameters as PubMed and looked at specific clinical studies as well as any review articles that pertained to COVID-19 diagnostic tests. The literature review was restricted to articles published in English. Authors used Google to search for reputable websites to attain background knowledge about the virus. The published papers that were chosen were written in 2020-2021.

Discussion

**Figure 1:**
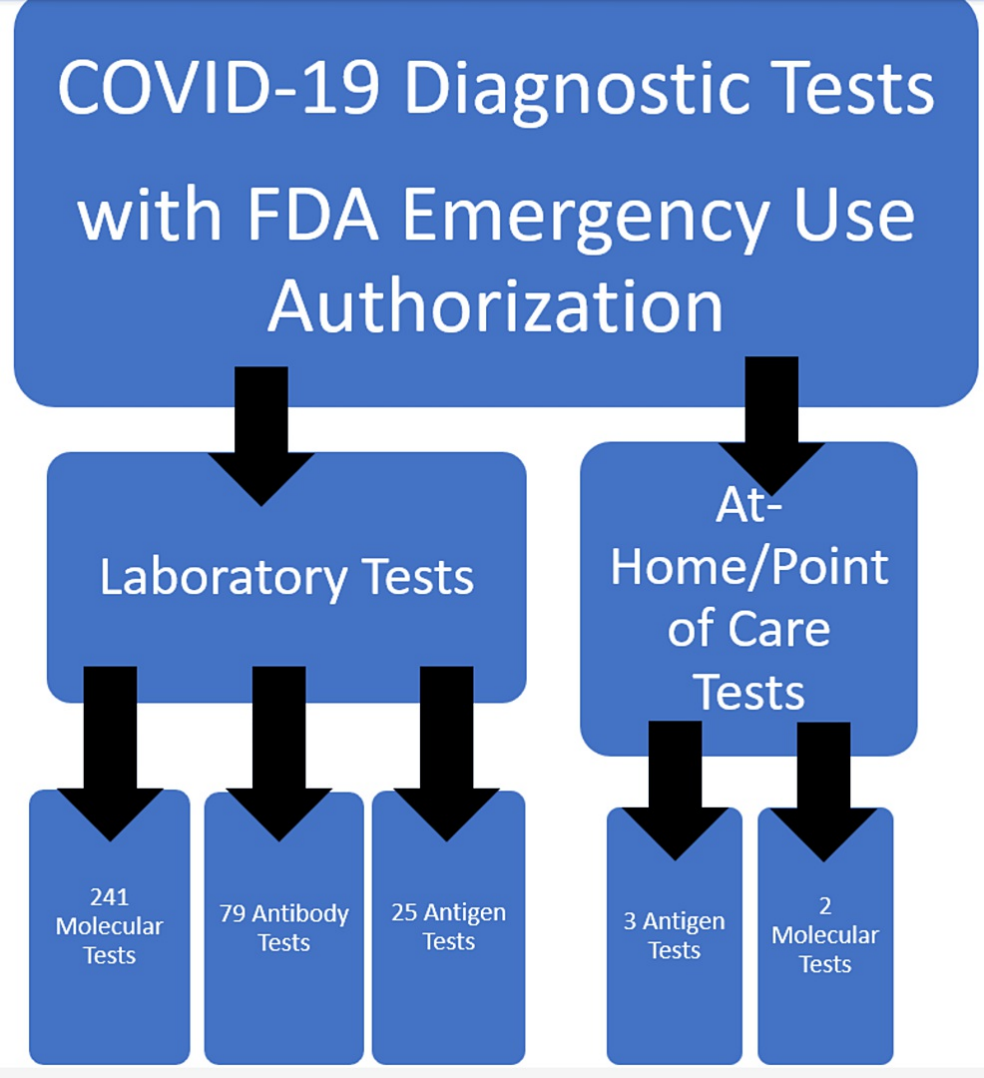
Laboratory and point-of-care testing for COVID-19

There are a variety of COVID-19 tests available for use. However, they are separated based on laboratory or point-of-care use. Figure 1 shows COVID-19 diagnostic tests with FDA EUA as of July 2022. All these tests have been given FDA EUA so they can be specifically utilized for the pandemic.

Regarding the laboratory tests, reverse-transcriptase polymerase chain reaction (RT-PCR) is generally used to determine the presence of SARS-CoV-2 infection inside the body of COVID-19 suspected patients. These NAAT assays detect RNA-dependent RNA polymerase, nucleocapsid, spike, and envelope genes as well as those that are present in the initial reading frame [11-13]. However, some tests have used antibodies or antigens to determine if viral nucleocapsid proteins are present. In antibody tests, the antibodies that are measured can either range from a combination of these or singularly - IgA, IgG, IgM, and total immunoglobulin. It has been noted that those tests that detect total immunoglobulin and/or IgG antibodies have shown higher accuracy than other specific antibody tests [11]. For antigen tests, viral SARS-CoV-2 protein is targeted from either nasopharynx, nasal cavity, or saliva, especially the viral nucleocapsid protein due to sheer abundance [11,14]. These antigen-based detection tests utilize chromatographic digital, microfluidic immunofluorescence, as well as lateral flow immunoassays and can be completed at home or in a laboratory [11,15]. Sensitivity and specificity have been included for each of these testing kits as it highlights the kit’s ability to detect the COVID-19 infection (sensitivity) and to exclude any other infections in the body (specificity). It has been noted that antigen tests are less sensitive than molecular tests as molecular tests amplify traces of viruses for detection. The differences in sensitivity can be attributed to false-negative results or missed detections of COVID-19. On the other hand, the specificity for these antigen tests is close to molecular tests, the difference attributed to higher false-positive results or incorrectly detecting a virus that is not present [16]. Table 1 summarizes the laboratory COVID-19 tests.

**Table 1:** Laboratory COVID-19 tests

Name of Test	Type of Test	Mechanism	FDA Approved?	Rapid Test?	Prescription Based?	Sensitivity (+)	Specificity (-)
Simplexa COVID-19 Direct Kit [17]	Molecular	Reverse Transcription Polymerase Chain Reaction (RT-PCR)	EUA	No	No	100%	100%
Cobas SARS-CoV-2 Test & Influenza A/B [18]	Molecular	RT-PCR	EUA	No	No	100% SARS-CoV-2; 98.4% Influenza A; 97.9% Influenza B	100% SARS-CoV-2; 96.5% Influenza A; 99.4% Influenza B
DxTerity COVID-19 Test [19]	Molecular	RT-PCR	EUA	No	No	97.3% Symptomatic Patients; 84.6% Asymptomatic Patients	90.0% Symptomatic Patients; 99.0% Asymptomatic Patients
Beckman Coulter’s Access SARS-Cov-2 IgG II [20]	Antibody	IgG, Paramagnetic particle chemiluminescent assay	EUA	No	No	100%	99.6%
ACON SARS-CoV-2 IgG/IgM Rapid Test [21]	Antibody	IgG & IgM, Lateral Flow assay	EUA	Yes	Yes	99.1%	98.2%
OmniPATH COVID-19 Total Antibody ELISA Test [22]	Antibody	Total Antibodies (IgG, IgA, IgM) - ELISA	EUA	Yes	No	100% (15 days post-symptoms)	100% (15 days post-symptoms)
Celltrion DiaTrust COVID-19 Antigen Rapid Test [23]	Antigen	Lateral Flow Immunoassay	EUA	Yes	Yes	93.33%	99.03%
BD Veritor System [24]	Antigen	Chromatographic Assay	EUA	Yes	No	89.6%	98.8%
Simoa SARS-CoV-2 N Protein Antigen Test [25]	Antigen	Paramagnetic microbead-based immunoassay	EUA	Yes	No	97.7%	100%

Nucleic Acid Amplification Tests (NAATs)

With the emergence of SARS-CoV-2, NAATs have been at the forefront of diagnosing an active and acute infection with the highest amount of sensitivity [26]. In accordance, there have been many developed diagnostic NAAT assays including RT-PCR, Loop-mediated isothermal amplification (LAMP), and CRISP assays [27,28]. These NAAT assays detect RNA-dependent RNA polymerase, nucleocapsid, spike, and envelope genes as well as those that are present in the initial reading frame [11-13]. There are currently 243 molecular-based COVID-19 tests that have been given emergency EUA from the FDA and the majority of these utilize RT-PCR [11]. 

Real-Time PCR Tests

To detect the COVID-19 virus molecularly, an analysis of the nucleic acids in a respiratory sample is completed. RT-PCR is the most commonly used laboratory method currently employed to detect COVID-19. This technique has been used to detect other viruses such as MERS-CoV and SARS-CoV [12,29,30]. Many primers and probes exist that can be used in RT-PCR to confirm the SARS-CoV-2 virus. RT-PCR tests can vary in length of time it takes to complete. Currently, there are two approaches that are used, a one-step and a two-step approach. The one-step approach involves the RT and DNA polymerase reaction being completed in the same reaction tube which is the preferred detection method for SARS-CoV-2 [27]. The two-step approach has the RT and DNA polymerase being detected in separate reaction tubes. One RT-PCR machine can analyze a range of one-to-many hundreds of samples in a given time frame. RT-PCR test results depend on the integrity of primers and probes used, sample collection methods, suited controls, and temperature control reliance. In addition, negative control and positive control are employed. Negative controls check against sample cross-contamination and positive controls ensure that the reagents, probes, and primers are accurate. The CDC recommends utilizing a human specimen control for better sample lysis and integrity of extraction reagents to markedly reduce false-negative results [11,31].

Simplexa COVID-19 Direct Kit, Cobas SARS-CoV-2 and DxTerity COVID-19 Test

The Simplexa COVID-19 direct assay RT-PCR diagnostic panel under EUA targets two genes, the S gene, and ORF1ab. RT-PCR assays typically involve more than one gene target to ensure the accuracy of COVID-19 diagnosis. The Simplexa COVID-19 direct assay is positive when both the ORF1ab and S gene targets are positive. If any of the two assays are negative, the results suggest a sample at a concentration near or below the detection limit of the test or a mutation in one of the gene target regions [17].

The Cobas SARS-CoV-2 and Influenza A/B protocol employ detection probes specific to SARS-CoV-2, sarbecovirus, influenza A, influenza B, and RNA internal control nucleic acid. Coronavirus, influenza A, B, and RNA internal Control detection probes are also labeled with unique fluorescent dyes acting as a reporter [18].

The DxTerity COVID-19 Test Collection kit protocol uses gene targets from the N (nucleocapsid) gene, E (envelope) gene, and ORF1ab region. Additionally, the human RNase P gene serves as a gene target internal and extraction control. The SalivaDirect RNA-extraction free, dualplex RT-PCR test protocol does not require saliva collection tubes with preservatives, or any special equipment for nucleic acid extraction. Saliva is pretreated with proteinase K followed by a heat inactivation step and then added to the test using reliable 2019-nCoV_N1 and RP (ribonuclease) primer and probe sets developed by the US CDC. The human RP probe was modified to use a different fluorophore so the primer/probe set could be used together in a dualplex setup to minimize the number of tests run to 1 [19].

Antibody-Based Testing

Antibody-based assays preferentially target SARS-CoV-2’s nucleocapsid protein as well as the spike protein, using either ELISA, chemiluminescence immunoassays (CLIA), or lateral flow immunoassays that have been assessed in various systemic studies [11,32-34]. In these tests, the antibodies that are measured can either range from a combination of these or singularly - IgA, IgG, IgM, and total immunoglobulin. It has been noted that those tests that detect total immunoglobulin and/or IgG antibodies have shown higher accuracy than other specific antibody tests [8]. Also, these serological tests should not be conducted during the earlier phases of the infection due to a high possibility of a false negative; it has been optimally recommended to test after three or four weeks of symptoms [11,35,36]. Although they are not commonly used to diagnose active infection, antibody tests are beneficial in epidemiology surveys whereby a specific infection rate can be found for a certain region or demographic [11]. A point of concern that these antibody tests have shown is of cross-reactivity with other human coronaviruses can lead to a high degree of false positivity [37,38]. The CDC is aware of such reactivity and has asked examinees to confirm their diagnosis with a second antibody assay, preferentially a different type [11,35].

There are 78 antibody diagnostic tests for SARS-CoV-2 listed under FDA’s EUA. However, testing is limited to laboratories certified under the Clinical Laboratory Improvement Amendments of 1988 (CLIA). The kits are differentiated based on what they are testing for and their methods. While some tests are testing for total antibody count, others are specific for IgG and IgM. The majority of these testing kits use either ELISA, Digital Lateral Flow, photometric immunoassay, lateral flow, or enzyme-linked fluorescent assay (ELFA). FDA has noted that these antibody tests are not validated to evaluate immunity from a SARS-CoV-2 infection or after vaccination. Furthermore, they should only be ordered by healthcare professionals who are knowledgeable about the limitations of such kits [39].

Beckman Coulter’s Access SARS-CoV-2 IgG II

Early in the outbreak, Beckman Coulter designed one of the first antibody testing kits to determine an adaptive immune response to SARS-CoV-2 [40]. It is used to qualitatively detect antibodies (IgG) against the virus’s spike proteins in human plasma using a paramagnetic particle, chemiluminescent immunoassay. In a clinical study, it had been highlighted that Beckman Coulter’s testing kit showed a 99.8% negative percent agreement (specificity) and a 100% positive percent agreement (sensitivity) - exemplifying its limited number of false positives and false negatives [40]. Like other antibody testing kits, individuals should test for antibody presence only after several days post-infection. For an active infection, a molecular diagnostic test should be utilized such as an RT-PCR test rather than an antibody test to further minimize any false negatives or positives. Additionally, a negative result can occur if not enough antibodies are present in the specimen. Beckman Coulter specifies that a negative or positive result should be confirmed with an antibody test from another manufacturer for the highest accuracy. Also, clinical performance has not been determined for all COVID-19 viral variants currently in circulation but is reflective of the variants found at the time of development including Alpha, Beta, Gamma, and Delta. It has been noted that interferences and erroneous results can arise due to an individual utilizing immunoglobulin-based treatments, those that are exposed to animals, and to those that produce excess rheumatoid factor or alkaline phosphatase [40].

ACON SARS-CoV-2 IgG/IgM Rapid Test

ACON Lab’s SARS-CoV-2 IgG/IgM Rapid Test is a prescription-based, lateral flow chromatographic immunoassay rapid test used to detect virus IgM and IgG antibodies in whole blood, plasma, and serum. The membrane is coated with anti-human IgG and IgM antibodies and when a specimen is added, these membrane antibodies will react to serum antibody presence [40]. In a study, ACON’s results from 73 individuals have been compared to RT-PCR results and it has been highlighted that IgG has a higher positive similarity to RT-PCR results in comparison to IgM. ACON’s sensitivity is 99.1%, relative specificity is 98.2% and accuracy is 98.5% [40].

In a cross-sectional study conducted in Ethiopia comparing the specificity, sensitivity, and agreement between three IgG/IgM rapid test kits - EGENE, CTK BIOTECK’s Onsite, and ACON - and RT-PCR testing, it has been noted the diagnostic sensitivity is 61.18%, 74.12%, 83.53%, and the specificity is 83.53%, 96.52%, 94.78%. Sisay et al. have highlighted that sensitivity for each of these tests increased after a week of clinical onset. It has been worth noting that these values do not correspond to the values provided by the manufacturers. However, these researchers did not collect additional specimens to confirm their results nor were able to determine the viral load amounts by way of nasal or throat swabs [41].

OmniPATH COVID-19 Total Antibody ELISA Test

OmniPATH’s COVID-19 Test is an ELISA-based kit intended to qualitatively detect the total amount of antibodies (IgM, IgG, and IgA) in human serum. Like other antibody testing kits, OmniPATH is used to identify individuals that have elicited an adaptive immune response due to SARS-CoV-2 - either recent or a former infection. Although the manufacturer has not provided the sensitivity of this particular test, they have noted that false positives may occur due to cross-reactivity from antibodies [22]. In addition, they have noted that total antibodies may not be detectable in less than two weeks post-infection. In an independent study, OmniPATH has shown nearly a 100% similarity in results compared to a NAAT with both IgG and IgM present in all samples. Also, it was determined that false positives could be due to HIV infection. As with other antibody tests, OmniPATH should not be used to determine acute infections and all results should be confirmed with an independent test - either antigen or nucleic acid based. In this assay, samples are added to the microwells embedded with antigen, if antibodies are detected they bind to the antigens and a second enzyme conjugate will catalyze a color reaction to show specific results [22].

Antigen-Based Testing

For these particular tests, viral SARS-CoV-2 protein is targeted from either nasopharynx, nasal cavity, or saliva, especially the viral nucleocapsid protein due to sheer abundance [11,14]. These antigen-based detection tests utilize chromatographic digital, microfluidic immunofluorescence, as well as lateral flow immunoassays and can be completed at home or in a laboratory [11,15]. The point-of-care tests (POCTs) and rapid diagnostics tests (RDTs) rely mostly on antigen or antibody detection and can be completed within minutes without laboratory involvement [11]. Currently, there are only a few POCTs that have been given EUA by the FDA. Although these POCTs and RDTs have an average specificity near 100%, they are less sensitive than NAATs with the average sensitivity being close to 55% with a maximum of 97% [11,22,40,41]. However, these simple-to-use POCTs are beneficial when NAATs are inaccessible or if time is limited. POCTs is most sensitive when there is a high viral load, especially during the earlier period of the illness [42,43]. It has been highlighted that WHO does not recommend POCTs for acute patient care testing if there are NAATs available, especially due to the lower sensitivity percentage seen with POCTs [14]. These POCTs have the ability to lead a patient away from their emergent diagnosis [11].

At this moment, under FDA’s EUA, there are 23 antigen diagnostic tests for SARS-CoV-2 [44]. Although the FDA has not approved these testing kits, they are released to the public and health officials in an attempt to alleviate the pandemic spread of SARS-CoV-2 [44]. For each of these tests, FDA has given specific limitations and conditions of authorization, especially in regard to certain approved testing laboratories. Some of them are prescription-ordered tests, while others are over the counter (OTC) or direct-to-consumer (DTC) home collection tests that are sent to laboratories for processing after sample collection [43]. Some of the lateral flow immunoassay antigen-based tests are Celltrion DiaTrust COVID-19 Ag Rapid Test, CareStart COVID-19 Antigen Test, Sofia SARS Antigen FIA, QuickVue SARS Antigen Test, Clip COVID Rapid Antigen Test. Furthermore, there are testing kits that rapidly detect SARS-CoV-2 and Flu A/B - BD Veritor System for Rapid Detection, Status COVID-19/Flu, Sofia 2 Flu + SARS Antigen FIA [44]. Some of these are described below. 

Celltrion DiaTrust COVID-19 Antigen Rapid Test - Lateral Flow Immunoassay

Celltrion DiaTrust’s COVID-19 Rapid Test is a lateral flow immunoassay, a prescription-based test used to detect SARS-CoV-2’s nucleocapsid and receptor-binding domains on the spike proteins. Individuals should swab their nasopharyngeal within seven days of symptom onset to retrieve a result. Positive results indicate the presence of antigens, however at times, with all POCTs, a second, different confirmatory test should be conducted. It is noted that positive results from this test do not eliminate any bacterial or viral co-infections and negative results should be treated as presumptive and confirmed with a molecular assay. As with the other rapid tests, Celltrion cannot differentiate between the SARS-CoV and SARS-CoV-2 strains. All results should be considered in context with the patient’s history as well as other results [45].

BD Veritor System - Chromatographic Assay/SARS-CoV-2, Flu A+B

BD’s Veritor System is used for the rapid detection of Flu A and B as well as SARS-CoV-2. It uses a chromatographic immunoassay to detect the nucleocapsid antigen of SARS-CoV-2, Influenza A or B [23]. Like the Sofia 2 Flu + SARS testing kit, it is able to detect both flu antigens as well as SARS antigens. It is a point-of-care test, however only intended under healthcare professional guidance [24]. All negative results received from this test should be treated as presumptive and should not be taken at face value without confirmation with a molecular-based assay or taking into account clinical signs and exposures [24]. For this test, the swab is inserted into a nostril and swirled in an extraction reagent tube. Following, some drops are added to the sample well and the test is run for 15 minutes. Then, the sample well is inserted into the BD Veritor Plus Analyzer for analysis and the results will appear on the display window [24]. Furthermore, Young et. al has stated that Veritor test allows for a more rapid SARS-CoV-2 testing by way of nasal swabbing. However, it has shown a less than 100% positive percent agreement when compared to PCR [46]. However, they also concluded that Sofia 2 Flu + SARS and Veritor have a high degree of agreement for SARS-CoV-2 detection [46]. Their results suggest that as the number of SARS-CoV-2-associated symptoms increases, the higher the sensitivity of the antigen-based test, allowing for a higher probability of an accurate result [46].

Simoa SARS-CoV-2 N Protein Antigen Test - Paramagnetic Microbead-Based Immunoassay

Quanterix Corp’s Simoa antigen test detects the presence of nucleocapsid protein from nasopharyngeal samples taken by an individual’s healthcare provider within 14 days of symptom onset. Quanterix is in the process of converting this test kit to a home-based sample collection kit for further rapid diagnosis. Simoa’s Antigen Test utilizes a microbead-based sandwich ELISA coupled with Simoa (single molecule array) technology. However, positive results cannot differentiate between SARS-CoV-2 and SARS-CoV or any co-infections. In a clinical study of 126 nasopharyngeal swab specimens from suspected SARS-CoV-2-infected individuals, RT-PCR and Simoa Protein Antigen Test were compared. It was found that Simoa has a 97.7% positive agreement and a 100% negative agreement with PCR. However, as the symptom onset days progressed, Simoa had a less than 100% positive agreement with PCR, specifically noting a 94.7% agreement when onset progressed to 8-14 days. In a separate study, Shan et al. highlight that Simoa SARS-CoV-2 can successfully and effectively detect SARS-CoV-2 antigen levels in both saliva and blood, leading to the future potential of a point of care, at-home sample kit. It has been noted, from their study, that Simoa immunoassay shows greater than 98% negative agreement and greater than 90% positive agreement with PCR molecular testing in regards to symptomatic, asymptomatic, and pre-symptomatic individuals during the first week of infection [25,47].

Point-of-Care Testing

At this moment, there are five at-home COVID-19 tests that have been given EUA from the FDA, with four that will be sold OTC without a prescription. Three of these are antigen-based tests, while the other two are molecular-based via isothermal amplification. These tests are revolutionizing the healthcare field as consumers now do not have to extended periods to retrieve their results. These new tests are more convenient, less expensive, and are essential for communities, workplaces, and schools to rapidly screen individuals. However, they have not gone through FDA’s extensive approval process and can be revoked if the FDA determines any ineffectiveness. These POCTs are significant in decentralized health care environments where PCR-based tests are inaccessible or with longer turnaround times [11,48]. Table 2 summarizes the point-of-care COVID-19 tests.

**Table 2:** Point-of-care COVID-19 tests

Name of Test	Type of Test	Mechanism	FDA Approved?	Rapid Test?	Prescription Based?	Sensitivity (+)	Specificity (-)
Ellume COVID-19 Home Test [49]	Antigen	Lateral Flow	EUA	Yes	No	95%	97%
Abbott BinaxNOW COVID-19 Ag Card Home Test [50]	Antigen	Lateral Flow	EUA	Yes	No	91.7%	100%
QuickVue At-Home OTC COVID-19 Test [51]	Antigen	Lateral Flow	EUA	Yes	No	83.5%	99.2%
Cue COVID-19 Test for Home and Over the Counter (OTC) Use [52]	Molecular	Isothermal Nucleic Acid Amplification	EUA	Yes	No	91.5%	95.7%
Lucira COVID-19 All-In-One Test Kit [53]	Molecular	Loop Mediated Isothermal Amplification	EUA	Yes	Yes	94%	98%

Although these POCTs show an accuracy rate of 80%, it has been highlighted that the antigen POCTs miss many asymptomatic infections, whereas the molecular-based POCTs are more effective in diagnosing an infected asymptomatic individual. However, the gold standard SARS-CoV-2 test is a PCR-based laboratory test that is more sensitive, and specific and can detect the virus at lower levels. A CDC study showed that Abbot’s antigen-based test found only 34% of infections in asymptomatic individuals [54]. Moreover, a complied review of 68 studies has found that rapid antigen-based tests identify nearly 58% of asymptomatic individuals and 72% of symptomatic individuals, so it is imperative that those using a POCT should continue wearing their masks, practice social distancing as well as other precautions. In fact, those with a negative result should identify any symptoms that may be associated with SARS-CoV-2 and look to re-test within 24-48 hours. It is imperative for individuals to double check their results with a laboratory-based PCR test as those are more accurate than POCTs [54].

Ellume COVID-19 Home Test - Antigen Based

The first at-home tests released can be purchased without a prescription. It is an antigen-based viral protein diagnostic test with results delivered to a smartphone application within 20 minutes. From a late-2020 clinical trial, it has been noted that Ellume can detect 96% of positive cases and 100% of negative cases from symptomatic individuals, however only 91% of positive cases and 96% of negative cases from asymptomatic individuals (FDA). It is likely infected individuals may receive a negative result due to the high rate of false negatives that this antigen-based test can produce, especially those who are pre-symptomatic or post-symptomatic. Symptomatic individuals have a greater statistical chance to receive an accurate diagnosis than those not showing any symptoms. It has been highlighted that asymptomatic individuals with negative results should receive additional testing to confirm the proper diagnosis. This inexpensive test will be widely available soon with a price point of $30. However, it cannot be used for children under the age of two. To obtain a test result, the nasal swab is inserted in each nostril until the swab cap or child adapter touches the nose with one’s smartphone three inches away and opened on the Ellume COVID-19 Home Test app. Once the results are received on the app, they can be shared with any healthcare professional [51,55].

Abbott’s BinaxNOW COVID-19 Ag Card Home Test - Antigen-Based

Abbott’s BinaxNOW COVID-19 Test is a home-use, lateral flow immunoassay used for the detection of COVID-19’s nucleocapsid protein antigen and conducted with assistance from a digital health solution, eMed. This home test uses an immunochromatographic membrane with specific SARS-CoV-2 antibodies and a control antibody to establish a test strip. To generally perform, a nasal swab is collected by the individual from both nostrils, six drops of reagent are added to the top well and after a few swab rotations, the test strip is brought into contact with the swab. After the card is sealed, a QR code is scanned and the results are shown on the card. The test results are visually shown within 15 minutes with pink/purple-colored positive resultant lines. This test can be shipped to a home address and with the guidance of a trained telehealth professional via a video call, the BinaxNOW test can be self-administered with results appearing on their NAVICA app. The test is prescribed by an individual’s healthcare provider within the first week of symptom onset and can only be performed under a telehealth provider’s supervision. This test is designed for individuals four years and older with parental assistance needed for younger children. The nucleocapsid protein antigen is detectable when the anterior nasal (nares) is swabbed. However, a negative result should be evaluated with an individual’s symptoms, exposures, and travel history [56,57]. Additionally, the test is sold in packs of two, so an individual can confirm their diagnosis by re-testing. It has been noted that these kits will be available in grocery and drug stores for purchase as OTC kits [54].

QuickVue At-Home OTC COVID-19 Test - Antigen-Based

Quidel’s QuickVue At-Home OTC COVID-19 Test is a point-of-care lateral flow immunoassay test designed to qualitatively detect the nucleocapsid protein antigen from SARS-CoV-2’s infected individuals within the first week of symptoms. At this moment, the FDA has approved the OTC purchase of this kit whereby these will be available in various drug stores across the US within weeks. Direct anterior nares self-swabbing can be completed if an individual is 14 years or older, however younger individuals must have an adult swab. From their various systemic reviews, QuickVue’s positive results show 83% similarity with PCR findings and 99% PCR similarity for negative results. Quidel has noted that individuals should re-test within 24-36 hours to ascertain confirmatory results. As with other POCTs, QuickVue cannot differentiate between SARS-CoV-2 and other coronaviruses. It is noted that positive results from this test do not eliminate any bacterial or viral co-infections and negative results should be treated as presumptive and confirmed with a molecular assay. In this test, a patient’s swab sample is added to the reagent tube and rolled three times within the reagent solution. Following, a test strip is added to the reagent tube whereby within 10 minutes results can be interpreted in accordance with the resultant key. The paper test strip is similar to a home pregnancy test with the results seen by a color change. All results should be considered in context with the patient’s history as well as other results. This test comes in a pack of two so individuals can confirm their diagnosis with a re-test [56,58].

Cue COVID-19 Test for Home and OTC Use - Molecular Based

Cue’s COVID-19 test detects SARS-CoV-2’s RNA and is one of the first at-home automated tests using molecular technology released by the FDA which can be purchased without a prescription. It has been noted that compared to a PCR laboratory-based test, Cue has a 97.4% agreement for positive cases and a 99.1% agreement for negative cases. Also, it has been noted that molecular-based tests are more accurate than those that use antigen technology. After a lower nasal swab is collected by the Cue Sample Wand, a nucleic acid application test (NAAT) is conducted on a rechargeable, palm-sized Cue Cartridge Reader and the results appear on the Cue Health app within 20 minutes. Cue’s OTC test has been highlighted as one of the more accurate, fast, and accessible at-home tests available to address the health circumstances produced by the pandemic. At this moment, these tests are being used for essential health facilities and K-12 schools and are being distributed by the U.S. Department of Defense and the U.S. Department of Health and Human Services. However, this test cannot be used for children under the age of two and cannot differentiate between SARS-CoV and SARS-CoV-2. Cue has emphasized that those individuals that receive a negative Cue result should confirm their diagnosis with a laboratory-performed molecular assay [57,59,60].

Lucira COVID-19 All-In-One Test Kit - Molecular Based

Lucira’s COVID-19 All-in-One Test Kit is a prescription-based, self-collecting, nasal swabbing test that is available for individuals 14 years and older, with a price of $50. Like Cue, it uses isothermal amplification to detect the RNA of SARS-CoV-2’s N gene by way of RT-LAMP amplification - a two-step reaction that is characterized by a cyclic and a non-cyclic phase. In the non-cyclic stage, RNA is converted into cDNA by reverse transcriptase and in the cyclic stage, the DNA polymerase amplifies the cDNA produced. For this to occur, the nasal swab contents from both nostrils are mixed with an elution buffer for RNA to be lysed and released. Within each chamber, the eluant is able to enter a fluidic molecule and begin the RT-LAMP reaction whereby a heating element is activated to gauge the reaction chamber. In this Test Unit, there are positive control chambers as well as chambers that specifically react with the RNA of SARS-CoV-2. Within 30 minutes, an amplification-RNA-positive color change is detected by a microprocessor, and by way of a diagnostic algorithm, a LED indicator is lit. The LED results can either represent invalid, positive, or negative [59,61].

Before releasing this test, Lucira Health determined that when testing cross-reactivity with 33 commensal organisms, none of the organisms were cross-reactive with Lucira’s test. Furthermore, they were able to test the reproducibility of the test by challenging untrained, intended users to utilize their test kit in order to determine if users are able to follow their directions and obtain expected results [61].

Limitations

Although the information is available for various tests, there is not enough comparative clinical information to conclude that a certain testing kit is far superior to the others. It is noted that NAATs are more sensitive and specific for acute infections, they are the gold standard when it comes to determining if an individual is infected with COVID-19. In the future, once more clinical, prospective and retrospective studies are completed we will be able to fully compare the efficacy and accuracy of each of these testing kits. Also, there are some published articles that highlight that COVID-19 antibodies may decrease to lower than the threshold post-infection, posing a problem for antibody-based tests in the future.

## Conclusions

Appropriate testing is of utmost importance in alleviating and downgrading a pandemic. Without specific or sensitive testing kits, the rate of COVID-19 cases would not have decreased as much as it has. The near-to-definite method to determine SARS-CoV-2 infection is by way of NAATs, specifically those using RT-PCR. It has been highlighted that antigen and antibody testing are rapid to determine infection. However, they should not be used in acute infections and should be confirmed with a NAAT. False positives, negatives, and cross-reactivity can occur with these testing kits, so it is imperative for an individual to be informed about all of these testing kits. With newer tests, we should see a shift towards more at-home, rapid assays that have high specificities as well as sensitivities.
